# Assessment of Wrist-Worn Activity Tracker Accuracy for Measuring Sedentary Behavior in Outpatients With Cardiovascular Disease: Validation Study

**DOI:** 10.2196/91104

**Published:** 2026-09-30

**Authors:** Koichi Naito, Kazuhiro P Izawa, Noriaki Maeda, Yuya Kasai, Haruna Tani, Misaki Kondo, Hajime Iwama

**Affiliations:** 1 Department of Physical Therapy, Faculty of Medical Science, Nagoya Aoi University, 3-40 Shioji-cho, Mizuho-ku, Nagoya, 4678610, Japan, 81 52-838-5026; 2 Iwama Cardiovascular and Dental Clinic for Prevention and Care, Kitakatsuragi-gun, Japan; 3 Department of Health Sciences, Graduate School of Medicine, Kobe University, Kobe, Japan; 4 Faculty of Sport Sciences, Waseda University, Saitama, Japan; 5 SapplyM, Inc., Tokyo, Japan

**Keywords:** sedentary behavior, wearable devices, consumer wearable, fitbit, validation study, bland-altman, concordance correlation coefficient, cardiac rehabilitation

## Abstract

**Background:**

Wearable monitoring of sedentary behavior is increasingly used in digital cardiovascular care, but the validity of consumer-grade wrist-worn trackers in older patients with cardiac issues is unclear.

**Objective:**

This study aimed to evaluate the agreement between sedentary time measured by a consumer-grade wrist-worn tracker (Fitbit Inspire 3; Fitbit LLC) and a research-grade waist-worn accelerometer (ActiveStyle Pro HJA-750C; Omron Healthcare Co, Ltd) used as the comparator, in Japanese outpatients with cardiac issues under free-living conditions.

**Methods:**

Fifty-one outpatients with heart failure (28/51, 55%) or ischemic heart disease (23/51, 45%; mean age 78.4, SD 9.0 years; 25/51, 49% male) simultaneously wore both devices for 7 consecutive days. Sedentary time was assessed within a standardized 12-hour daytime window (9 AM to 9 PM) at 1-minute epochs. Agreement was examined using Pearson correlation, Bland-Altman analysis (after confirming normality of the paired differences), Lin concordance correlation coefficient (CCC), and Cohen kappa for distribution-based tertile classification. A constant bias correction was applied, with leave-one-out cross-validation (LOOCV) used as an internal-consistency check on the stability of the sample mean bias. Moderators of the bias were explored using multivariable regression.

**Results:**

Mean sedentary time within the 12-hour window was 497.9 (SD 86.5) minutes for the comparator and 559.9 (SD 84.3) minutes for the Fitbit. The devices were strongly correlated (*r*=0.905, 95% CI 0.838‐0.945; *P*<.001). The Fitbit showed a significant fixed bias of 62.0 minutes (95% CI 51.5‐72.5; *P*<.001) with no proportional bias (slope −0.03; *P*=.67); the 95% limits of agreement were wide (−11.3 to 135.2 minutes). Uncorrected concordance was moderate (CCC=0.71, 95% CI 0.57‐0.80) and improved to high after subtracting the 62-minute bias (CCC=0.904, 95% CI 0.84‐0.94); LOOCV indicated that this in-sample improvement was internally stable (out-of-sample CCC=0.90). No candidate factor (sex, age, diagnosis, and musculoskeletal disorder) significantly moderated the bias (model *P*=.12). Tertile classification showed moderate agreement (κ=0.588, 95% CI 0.405‐0.772; 37/51, 72.5% agreement), with all misclassifications between adjacent categories.

**Conclusions:**

In older Japanese outpatients with cardiac issues, a consumer-grade wrist-worn tracker overestimated sedentary time by approximately 62 minutes within a standardized 12-hour daytime window compared with a waist-worn comparator, with fixed but no proportional bias. A simple constant correction markedly improved group-level concordance (cross-validated CCC≈0.90), supporting calibrated use of consumer wearables for scalable monitoring in ambulatory cardiac care. However, wide limits of agreement constrain individual-level precision, and the population-specific correction factor requires independent validation before transfer to other settings.

## Introduction

Sedentary behavior (SB)—any waking behavior performed in a sitting, reclining, or lying posture with an energy expenditure of ≤1.5 metabolic equivalents (METs)—has become recognized as a powerful, modifiable risk factor for cardiovascular morbidity and mortality [1,2]. A 2024 systematic review and dose-response meta-analysis of 19 prospective cohorts comprising 1,473,354 individuals and 60,526 cardiovascular events found that participants in the highest SB category had a 30% higher risk of fatal and nonfatal cardiovascular disease (CVD) compared with the lowest category (relative risk [RR] 1.29, 95% CI 1.22‐1.37). Each additional hour of sedentary time was associated with a 5% increase in CVD risk (RR 1.05, 95% CI 1.02‐1.07). The dose-response curve exhibited a J-shaped pattern, and replacing one hour of sitting with light-intensity physical activity (LPA) lowered CVD risk by 20% (RR 0.84, 95% CI 0.73-0.97) [3]. These quantitative estimates underline the clinical importance of reducing and breaking up prolonged sitting [4,5]. Global public health guidance also explicitly highlights reducing sedentary behavior alongside increasing physical activity across the life course [6].

A recent Japanese study of 92 outpatients undergoing cardiac rehabilitation measured with a triaxial waist-worn accelerometer reported average SB of 663 (SD 118) minutes/day (≈11 hours) and moderate-to-vigorous physical activity (MVPA) of just 25 minutes/day; men sat 64 minutes/day more than women and performed 22% less light-intensity activity [7]. Such findings underscore the importance of sex-specific strategies to break up SB in Asian settings where population aging is pronounced [8,9].

Emerging research emphasizes that wearable device–based measurement and intervention targeting sedentary behavior are critical components of secondary prevention strategies in patients with coronary artery disease (CAD). Continuous monitoring of sedentary time allows for the identification of at-risk individuals, while targeted interventions informed by wearable analytics can support behavioral modification and contribute to improved cardiovascular outcomes [10-12]. Research-grade triaxial accelerometers, such as the waist-worn ActiveStyle Pro (Omron Healthcare Co., Ltd.), have demonstrated high validity for estimating METs compared with the Douglas bag method and are considered effective for sedentary behavior measurement [13]. Nevertheless, maintaining compliance with device wear over prolonged monitoring periods remains a challenge.

Inexpensive wrist-worn consumer trackers—most prominently the Fitbit (Fitbit LLC) series—are ubiquitous, integrate seamlessly with smartphone dashboards, and are attractive for remote monitoring [14,15]. Yet their proprietary algorithms were engineered primarily for step detection; recent investigations in healthy adults show systematic overestimation of SB stemming from arm movements [16-18], and wrist placement cannot directly capture postural transitions that define sedentary behavior, requiring inference from movement patterns that can be prone to misclassification [19]. Their validity in clinical populations therefore remains uncertain [20].

Thus, we aimed to evaluate the agreement between sedentary time estimates derived from a consumer-grade wrist-worn device (Fitbit) and a research-grade waist-worn accelerometer (ActiveStyle Pro) in Japanese outpatients with cardiac issues under free-living conditions. We used Bland-Altman plots to characterize systematic and proportional bias [21], calculated Lin concordance-correlation coefficient (CCC) [22], and assessed tertile classification agreement using Cohen kappa [23]. Prior validation studies [24-28] comparing wrist-worn Fitbits with research-grade monitors report mixed bias for sedentary time, with differences ranging from modest underestimation to overestimation depending on the comparator (ActiGraph; ActiGraph LLC vs activPAL; PAL Technologies Ltd), wear location, population, and data processing. Accordingly, we hypothesized the presence of systematic bias without prespecifying its direction and used Bland-Altman analysis to characterize both fixed and proportional components, also testing whether a constant offset improved concordance.

By clarifying the magnitude and nature of measurement error in this clinically important population—and situating our results alongside emerging Japanese data highlighting high SB levels and sex differences [7]—our findings aim to guide researchers selecting wearable sensors for SB trials and to assist clinicians interpreting tracker-derived metrics when delivering behavior-change counseling in ambulatory cardiac care.

## Methods

### Study Design

This cross-sectional validation study was conducted at a single cardiology outpatient clinic in Nara Prefecture, Japan, between October 2022 and March 2023.

### Patient Characteristics

Fifty-one consecutive outpatients with CAD or heart failure who were enrolled in the outpatient cardiac rehabilitation program were recruited for this study. Inclusion criteria were as follows: (1) diagnosed with ischemic heart disease or heart failure, (2) aged ≥18 years, (3) able to walk independently without assistive devices, (4) willing to wear both activity monitors simultaneously for 7 consecutive days, and (5) capable of providing informed consent. Exclusion criteria included (1) acute decompensated heart failure requiring hospitalization, (2) unstable angina or recent acute coronary syndrome within 4 weeks, (3) severe cognitive impairment precluding informed consent, (4) skin conditions preventing device attachment, and (5) planned hospitalization or major procedure during the monitoring period.

Demographic and clinical data were extracted from medical records, including age, sex, height, weight, BMI, primary cardiac diagnosis (heart failure vs ischemic heart disease), comorbidities (hypertension, dyslipidemia, diabetes mellitus, respiratory disease, renal disease, cerebrovascular disease, musculoskeletal disorders, malignancy), and current medications (beta-blockers, hyperpolarization-activated cyclic nucleotide [HCN]-gated channel blockers, angiotensin-converting enzyme [ACE] inhibitors or angiotensin receptor blockers [ARBs], mineralocorticoid receptor antagonists, angiotensin receptor–neprilysin inhibitor [ARNI], sodium-glucose cotransporter-2 [SGLT2] inhibitors, calcium channel blockers, nitrates, diuretics, statins). New York Heart Association (NYHA) functional class was not collected because the cohort included both patients with heart failure and patients with ischemic heart disease, for whom NYHA classification was not uniformly applicable. Musculoskeletal disorders were extracted from the medical records as an overall comorbidity category. No participant had a documented upper-limb musculoskeletal disorder that was expected to limit arm movement or affect wrist-worn device measurements.

### Sedentary Time

Participants simultaneously wore 2 activity monitors for 7 consecutive days: a research-grade triaxial accelerometer (ActiveStyle Pro HJA-750C) positioned on the waist and a consumer-grade wrist-worn tracker (Fitbit Inspire 3) on the nondominant wrist. The ActiveStyle Pro has been validated for measuring METs against the Douglas bag method [13] and its comparability with other commonly used activity monitors has been examined in free-living contexts [29,30]. The ActiveStyle Pro is widely used in sedentary behavior research [7,31]. The Fitbit Inspire 3 represents the latest generation of consumer wearables with integrated smartphone connectivity.

Participants were instructed to wear both devices continuously during waking hours (from awakening until bedtime) but were permitted to remove them during water-based activities (bathing and swimming). Detailed written and verbal instructions were provided regarding proper device placement and daily charging procedures. Participants maintained a simple daily log recording wake and sleep times and any periods when devices were removed.

Sedentary time was defined as any waking behavior performed in a sitting, reclining, or lying posture with an energy expenditure ≤1.5 METs, consistent with international consensus definitions [1]. For analysis, we extracted sedentary time data from both devices for the period between 9 AM and 9 PM to standardize the measurement window across participants and minimize the influence of sleep-related recordings. Data were recorded at 1-minute epochs. Daily sedentary time was calculated as the sum of all minutes classified as sedentary behavior within the standardized 12-hour measurement window.

To be included in the final analysis, participants were required to have at least 4 valid days of data, defined as days with ≥10 hours of concurrent wear time for both devices within the 9 AM to 9 PM measurement window. Mean sedentary time within the standardized 12-hour window across all valid days was calculated for each participant for both the ActiveStyle Pro and Fitbit devices.

### Fitbit Data Extraction and Sedentary Classification

Minute-level activity data were retrieved from the Fitbit Web API (Activity Intraday time series), which returns data at 1-minute intervals. As the intensity index for each epoch, we used the MET value returned with the calories resource. Because the Fitbit API expresses this value as METs multiplied by 10 (eg, a returned value of 15 corresponds to 1.5 METs), we defined an epoch as sedentary when the returned MET value was below 15 (ie, <1.5 METs) and the step count for the same minute was zero. This operationalization aligns the Fitbit-derived classification with the international ≤1.5 MET definition of sedentary behavior applied to the ActiveStyle Pro comparator. We emphasize, however, that this classification is an estimate based on the activity intensity and step information provided by the device and does not constitute a direct measurement of posture; this difference in how “sedentary” is operationalized between the 2 devices is itself a likely contributor to the observed bias.

### Nonwear Time

Minutes for which the Fitbit returned no detected heart rate were treated as nonwear and excluded from analysis. For the ActiveStyle Pro, a period of 60 or more consecutive minutes of zero counts was treated as nonwear. Valid days required at least 10 hours of concurrent wear time for both devices within the 9 AM to 9 PM window, and participants with at least 4 valid days were retained, as described above.

### Daytime Sleep and Naps

Participants were instructed to record any daytime napping in their paper daily log. No participant reported a daytime nap during the monitoring period; therefore, daytime sleep did not contribute to the sedentary tallies of either device, and any device-specific differences in nap handling could not have confounded the present comparison.

### Fitbit Companion App Configuration

The wrist-placement setting in the Fitbit companion app was configured to match the actual placement of the device on the participant’s nondominant wrist, ensuring that the device’s motion-processing sensitivity corresponded to the physical wear location and removing altered handedness settings as a potential source of systematic error.

### Statistical Analysis

Continuous variables are presented as mean (SD) or median (IQR) according to the normality of their distribution, assessed with the Shapiro-Wilk test. Categorical variables are presented as frequencies and percentages. Because n <100, percentages are reported without decimal places, and absolute numbers are reported alongside percentages.

Agreement between the ActiveStyle Pro comparator and the Fitbit was evaluated with several complementary analyses. Pearson correlation quantified the strength of the linear relationship. Bland-Altman analysis was used to assess systematic bias and to calculate the 95% limits of agreement [21]. Because the construction of parametric limits of agreement assumes that the paired differences are normally distributed, we first verified this assumption with the Shapiro-Wilk test applied to the differences. Fixed bias was tested with a one-sample *t* test of the paired differences, and proportional bias was examined by regressing the differences on the means of the two measurements.

Lin CCC was calculated to evaluate overall agreement, combining precision and accuracy, with 95% CIs obtained by bootstrap resampling [22]. The CCC ranges from −1 to +1, with values closer to 1 indicating better agreement. Categorical agreement was assessed by classifying each participant into distribution-based tertiles (lower, middle, and upper third) of sedentary time separately for each device and computing the Cohen kappa coefficient for the resulting categories [23].

Where Bland-Altman analysis indicated significant fixed bias, we conducted a secondary analysis subtracting the mean bias from the Fitbit values and recomputing agreement. As an internal consistency check on the stability of the sample mean bias, we recomputed the correction using leave-one-out cross-validation (LOOCV): for each participant, the mean bias was estimated from the remaining participants and applied to that held-out participant, and the concordance of the cross-validated, bias-corrected Fitbit values with the comparator was then assessed. As a sensitivity analysis, we repeated this resampling procedure using repeated random 50/50 splits (Monte Carlo cross-validation, 2000 repetitions).

As an exploratory, hypothesis-generating analysis of whether the bias was moderated by participant characteristics, we fitted a single multivariable linear regression of the individual difference (Fitbit−ActiveStyle Pro) on sex, age, primary cardiac diagnosis (heart failure vs ischemic heart disease), and presence of a musculoskeletal disorder. Because the study was not powered for subgroup detection, a single adjusted model was used rather than multiple stratified tests.

All analyses were performed in R version 4.1.3 (R Foundation for Statistical Computing); statistical significance was set at *P*<.05.

### Ethical Considerations

This study was approved by the Ethics Review Committee of Nagoya Aoi University (formerly Nagoya Women’s University; approval number 2022‐33) and was conducted in accordance with the principles of the Declaration of Helsinki. Written informed consent was obtained from all participants prior to enrollment. To protect privacy and confidentiality, data collection and data analysis were carried out by different personnel, and analyses were performed on deidentified data. No compensation was provided to participants. No images or other materials permitting identification of individual participants are included in this manuscript or its supplementary files.

## Results

### Patient Characteristics

A total of 51 outpatients (25/51, 49% male; mean age 78.4, SD 9.0 years) were enrolled in the study. Participant characteristics are summarized in Table 1. The mean BMI was 23.8 (SD 4.6) kg/m². The primary cardiac diagnosis was heart failure in 55% (28/51) of participants and ischemic heart disease in 45% (23/51). The cohort had a high burden of cardiovascular risk factors and comorbidities: hypertension (44/51, 86%), dyslipidemia (36/51, 71%), diabetes mellitus (12/51, 24%), renal disease (20/51, 39%), and musculoskeletal disorders (18/51, 35%). Most participants were receiving evidence-based cardiovascular medications, including statins (34/51, 67%), ARNI (31/51, 61%), beta-blockers (24/51, 47%), calcium channel blockers (21/51, 41%), and SGLT2 inhibitors (31%, 16/51; Table 1).

**Table 1.** Baseline characteristics of study participants (N=51).

Characteristics	Value
Demographics	
Age (years), mean (SD)	78.4 (9.0)
Sex (male), n (%)	25 (49)
Height (cm), mean (SD)	158.0 (8.6)
Weight (kg), mean (SD)	59.3 (12.8)
BMI (kg/m²), mean (SD)	23.8 (4.6)
Primary cardiac diagnosis, n (%)	
Heart failure	28 (55)
Ischemic heart disease	23 (45)
Comorbidities, n (%)	
Hypertension	44 (86)
Dyslipidemia	36 (71)
Diabetes mellitus	12 (24)
Renal disease	20 (39)
Musculoskeletal disorders	18 (35)
Respiratory disease	6 (12)
Cerebrovascular disease	5 (10)
Malignancy	4 (8)
Medications, n (%)	
Statins	34 (67)
ARNI^a^	31 (61)
Beta-blockers	24 (47)
Calcium channel blockers	21 (41)
SGLT2^b^ inhibitors	16 (31)
ARB^c^	11 (22)
HCN^d^ channel blockers	11 (22)
Mineralocorticoid receptor antagonists	7 (14)
ACE^e^ inhibitors	3 (6)
Nitrates	2 (4)
Diuretics	2 (4)

a ARNI: angiotensin receptor–neprilysin inhibitor.

b SGLT2: sodium-glucose cotransporter-2.

c ARB: angiotensin receptor blocker.

d HCN: hyperpolarization-activated cyclic nucleotide–gated.

e ACE: angiotensin-converting enzyme.

All participants successfully completed the 7-day monitoring period with both devices. The median number of valid measurement days was 7 (IQR 7‐7), with all participants achieving at least 4 valid days of concurrent device wear. Mean daily wear time within the standardized measurement window (9 AM to 9 PM) was 11.8 (SD 0.4) hours, indicating high compliance with the monitoring protocol.

### Agreement Between ActiveStyle Pro and Fitbit Measurements

Mean sedentary time within the standardized 12-hour window (9 AM to 9 PM) was 497.9 (SD 86.5) minutes for the ActiveStyle Pro comparator and 559.9 (SD 84.3) minutes for the Fitbit. A strong positive correlation was found between the two devices (Pearson *r*=0.905, 95% CI 0.838‐0.945; *P*<.001; Figure 1).

The paired differences were normally distributed (Shapiro-Wilk W=0.97; *P*=.16), supporting the use of parametric limits of agreement. Bland-Altman analysis revealed a significant fixed bias of 62.0 minutes (95% CI 51.5‐72.5; *P*<.001), indicating that the Fitbit systematically overestimated sedentary time relative to the comparator. The 95% limits of agreement were wide, ranging from −11.3 to 135.2 minutes, indicating substantial interindividual variability in the magnitude of disagreement. Regression of the differences on the means showed a significant intercept of 76.4 minutes (95% CI 7.7‐145.2; *P*=.03) and a nonsignificant slope of −0.03 (95% CI −0.16 to 0.10; *P*=.67), confirming fixed bias without proportional bias; that is, the degree of overestimation was approximately constant across the range of sedentary time (Figure 2).

**Figure 1.**
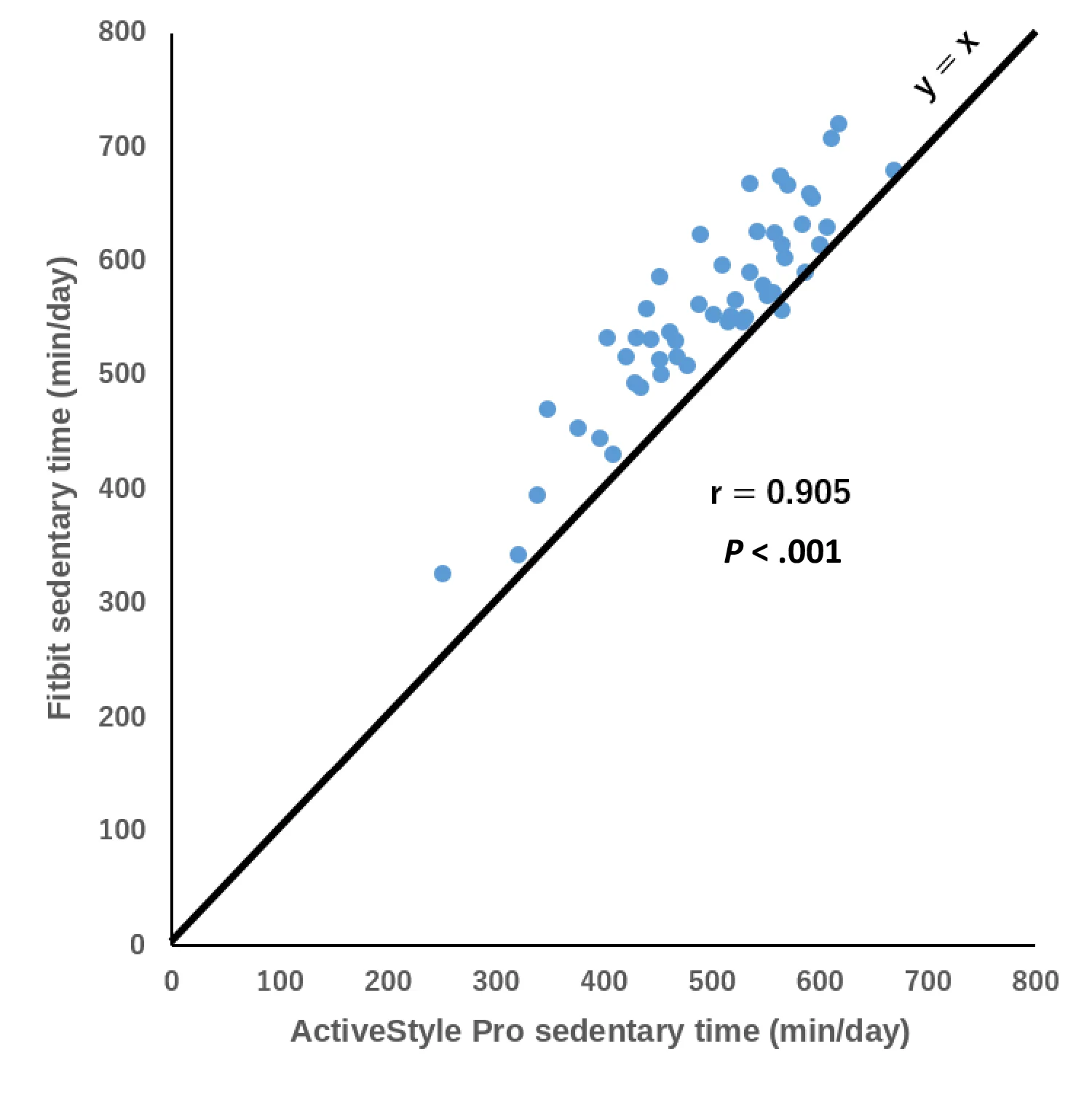
Scatter plot of mean sedentary time within the standardized 12-hour window (9 AM to 9 PM) measured by Fitbit Inspire 3 vs ActiveStyle Pro (HJA-750C) in outpatients with cardiac issues (N=51). Each point represents one participant’s mean sedentary time across valid days. Pearson correlation coefficient is shown.

**Figure 2.**
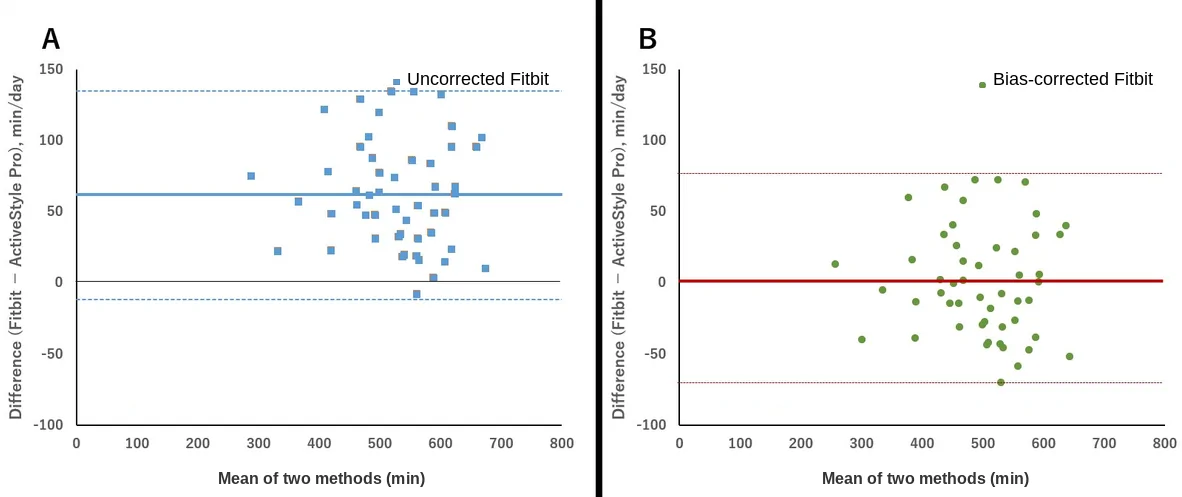
Bland-Altman plots comparing sedentary time between Fitbit Inspire 3 and ActiveStyle Pro (HJA-750C) in outpatients with cardiac issues (N=51). The y-axis shows the difference (Fitbit−ActiveStyle Pro) and the x-axis shows the mean of the two methods. The horizontal line at y=0 indicates zero difference (perfect agreement). Solid lines indicate the mean difference (bias), and dashed lines indicate the 95% limits of agreement. A: uncorrected Fitbit estimates. B: bias-corrected Fitbit estimates obtained by subtracting 62 minutes per 12-hour window (9 AM to 9 PM) from Fitbit values.

When sedentary time was classified into distribution-based tertiles computed separately for each device (ActiveStyle Pro boundaries: lower third <458 minutes, middle third 458‐549 minutes, upper third >549 minutes; Fitbit boundaries: lower third <533 minutes, middle third533‐593 minutes, upper third >593 minutes), the Cohen kappa coefficient was 0.588 (95% CI 0.405‐0.772), indicating moderate categorical agreement. The overall agreement rate was 72.5% (37/51 participants), with an agreement of 82.4% (14/17), 58.8% (10/17), and 76.5% (13/17) in the lower, middle, and upper tertiles, respectively. All 14 misclassifications (27.5%) occurred between adjacent tertiles, with no participant misclassified by two or more categories. The middle tertile showed the lowest agreement, indicating that classification was most difficult near category boundaries. The mean absolute difference in measured sedentary time among misclassified cases was 70.3 (SD 49.7) minutes.

After subtracting 62 minutes from the Fitbit values to correct for the fixed bias, regression of the corrected differences on the means showed no significant fixed bias (intercept=13.6 minutes, 95% CI −51.3 to 78.4; *P*=.68) while proportional bias remained absent (slope=−0.03; *P*=.67). The in-sample CCC improved from 0.71 (95% CI 0.57‐0.80) to 0.904 (95% CI 0.84‐0.94). To assess the internal stability of the in-sample correction, we used LOOCV: the out-of-sample CCC was 0.901, essentially identical to the in-sample value (shrinkage=0.004), and the per-fold bias estimate was highly stable (range 60.5‐63.4 minutes; SD 0.7). Repeated random 50/50 splits (Monte Carlo cross-validation) yielded a mean out-of-sample concordance of 0.89 (95% interval 0.81‐0.94). This reflects the stability of the sample mean within the present dataset and does not by itself establish that the correction factor generalizes to other samples (see Study limitations). Notwithstanding this group-level improvement, the limits of agreement for the cross-validated corrected values remained wide (approximately −74.7 to 74.7 minutes), indicating that correction reduces systematic bias at the group level but does not resolve individual-level imprecision (Figure 2B).

In the exploratory multivariable model of the individual difference (Fitbit−ActiveStyle Pro), none of the candidate characteristics significantly moderated the bias. The adjusted estimates were −13.9 minutes for male versus female sex (95% CI −34.4 to 6.5; *P*=.18), +0.7 minutes per additional year of age (95% CI −0.5 to 1.9; *P*=.25), +17.4 minutes for heart failure versus ischemic heart disease (95% CI −3.7 to 38.5; *P*=.10), and −6.7 minutes for the presence of a musculoskeletal disorder (95% CI −28.0 to 14.5; *P*=.53). The overall model explained little of the variance and was not statistically significant (*R*²=0.15; model *P*=.12). Although heart failure versus ischemic heart disease and older versus younger age appeared associated with the bias when examined in isolation, neither remained significant after mutual adjustment, consistent with confounding between age and diagnosis and with multiple comparisons. However, this model was underpowered (N=51; model *P*=.12; *R*²=0.15), and these null results should not be read as evidence that clinically relevant subgroup differences are absent. In particular, the adjusted estimate for heart failure versus ischemic heart disease (+17.4 minutes, 95% CI −3.7 to 38.5 minutes) remains compatible with a difference large enough to matter relative to the overall 62-minute bias. We therefore regard these analyses as hypothesis-generating only and do not conclude that a single population-level correction is definitively adequate across clinically important subgroups (see Study limitations).

## Discussion

### Summary of Main Findings

This study evaluated the validity of a consumer-grade wrist-worn activity tracker (Fitbit Inspire 3) for measuring sedentary time in Japanese outpatients with cardiac issues, using a research-grade waist-worn accelerometer (ActiveStyle Pro) as the comparator. We found that the Fitbit systematically overestimated sedentary time by approximately 62 minutes within a standardized 12-hour daytime window (9 AM to 9 PM; values are therefore not full-day estimates), with clear evidence of fixed bias but no proportional bias across the range of sedentary behavior. Although uncorrected agreement was only moderate, applying a simple constant correction substantially improved concordance between devices. Taken together, these findings indicate that consumer-grade wrist-worn devices can provide clinically meaningful information on sedentary behavior in cardiac outpatients with cardiac issues when their systematic measurement error is explicitly acknowledged and appropriately calibrated.

### Comparison With Previous Validation Studies

Our finding that the Fitbit overestimated sedentary time by approximately 62 minutes within the standardized 12-hour daytime window (9 AM to 9 PM) contrasts with some previous validation studies [24,25] that reported underestimation of sedentary time by wrist-worn devices. This discrepancy warrants careful consideration and may be explained by several factors related to our specific study population and context, while also revealing important insights into the mechanisms underlying device measurement error.

### Population-Specific Movement Patterns

Population characteristics likely play a crucial role in explaining the direction of bias. Our study enrolled older outpatients with cardiac issues (mean age 78.4, SD 9.0 years) with heart failure or ischemic heart disease, whereas many previous validation studies examined younger, healthier adults. The mean sedentary time observed with the ActiveStyle Pro comparator was 498 minutes within the standardized 12-hour window (9 AM to 9 PM; approximately 8.3 hours). Extrapolated across typical waking hours, this is broadly consistent with the approximately 11 hours per day reported in recent Japanese data on rehabilitation patients with cardiac issues, although our measurement was confined to the standardized window. This underscores both the clinical relevance of accurate sedentary behavior measurement in this population and the distinctive activity profile that may influence device performance.

Older patients with cardiac issues may exhibit distinct movement patterns during daily activities. Specifically, even when standing or performing light activities such as cooking, housework, or slow walking, these patients may have minimal arm displacement due to age-related changes in gait, cardiovascular limitations restricting vigorous arm swing, or cautious movement patterns to avoid symptoms. Consequently, periods of standing or light activity with stationary arms may be misclassified by the wrist-worn device as sedentary time, leading to overestimation.

In contrast, previous studies [24,25] reporting underestimation may have examined populations where wrist movements during sedentary activities (eg, typing, using smartphones or tablets while sitting, and hand gestures during conversation) were sufficient to trigger activity classification, resulting in sedentary time being counted as active time. Furthermore, participants in these studies had mean ages of approximately 20 and 68 years, which are younger than those in the current study, suggesting that age-related differences in movement patterns may partly explain this discrepancy.

Taken together, these considerations suggest that the overestimation observed in older outpatients with cardiac issues reflects predictable interactions between age-related movement patterns and the limitations of wrist-based sensing, underscoring the need for population-specific validation when applying consumer wearables in clinical cardiac care.

### Device Design and Algorithmic Factors

The consistent 62-minute overestimation reflects fundamental differences between wrist-worn and waist-worn device designs. Wrist placement cannot directly measure postural transitions (sitting-to-standing and lying-to-sitting) that are the defining feature of sedentary behavior. Instead, wrist devices must infer posture from arm movement patterns—an indirect approach prone to misclassification [19,25]. When individuals stand still or perform light activities with minimal arm displacement, the wrist device interprets the lack of movement as sedentary behavior, regardless of actual posture. Conversely, waist-worn devices like the ActiveStyle Pro capture whole-body movement and postural changes more directly through detection of trunk position and acceleration patterns.

Proprietary algorithms in consumer devices are optimized primarily for step counting and moderate-to-vigorous activity tracking, the metrics most valued by general consumers, rather than sedentary time classification. The Fitbit Inspire 3 represents a newer generation of consumer wearables with updated algorithms compared to devices examined in earlier studies. The threshold and decision rules for classifying sedentary versus active states may have been modified across device generations and may not be optimized for populations with limited mobility or distinct movement signatures.

### Comparator Device and Methodological Considerations

Device and algorithm differences across validation studies must be considered. Our comparator was the ActiveStyle Pro, a waist-worn triaxial accelerometer validated against metabolic measurements (Douglas bag method), whereas previous studies often used the ActiGraph or activPAL (a thigh-worn inclinometer). Different comparator devices use distinct classification approaches, posture-based (activPAL, using inclinometry) versus movement-based (ActiGraph and ActiveStyle Pro, using acceleration thresholds), which may influence the magnitude and direction of observed bias. Direct comparison across validation studies is complicated by this methodological heterogeneity.

Additionally, activity context and measurement protocol may contribute to differences across studies. We measured sedentary time during a standardized 12-hour daytime window (9 AM to 9 PM) in free-living conditions. In older patients with cardiac issues, this window likely captures prolonged periods of sitting while watching television, reading, or resting—activities performed with relatively stationary arms. If healthy adults in previous studies engaged in more dynamic activities with frequent arm movements during nonsedentary time, wrist devices might better distinguish active from sedentary periods in those populations.

### Implications of the Absence of Proportional Bias

Importantly, the absence of proportional bias in our data, confirmed by the nonsignificant regression slope (*P*=.67), indicates that the magnitude of overestimation remains constant regardless of whether individuals have low or high sedentary time. This finding has both mechanistic and practical implications.

Mechanistically, the consistent bias suggests that overestimation stems from systematic misclassification of specific activity types (low-movement standing and light activities) rather than from device calibration errors that would vary with activity volume. If the bias were due to gain or sensitivity errors, we would expect proportional bias where the error increases with higher sedentary time. Instead, the fixed bias pattern indicates that a relatively constant duration of nonsedentary activities (approximately 62 minutes within the 12-hour window; 9 AM to 9 PM) is being misclassified as sedentary across all participants.

Practically, this finding means that a simple constant correction can substantially improve agreement, as evidenced by the improvement in CCC from 0.71 to 0.904 after subtracting 62 minutes. This correction approach would not be appropriate if proportional bias were present.

### Context Dependency of Device Validity

The mixed findings across validation studies, including our own, underscore a critical point: wearable device validity is population-specific and context-dependent. A device validated in one population cannot be assumed to perform equivalently in another, particularly when movement patterns and activity repertoires differ substantially. This context-dependency extends beyond population characteristics to include cultural factors (eg, floor-sitting in some Asian populations), occupational demands, and the specific activities that comprise daily routines.

### Research Applications

For research applications, our results suggest that Fitbit devices should not be used interchangeably with research-grade accelerometers for primary outcome assessment without applying appropriate corrections or acknowledging measurement limitations. The moderate uncorrected CCC (0.71) indicates insufficient agreement for studies requiring precise absolute sedentary time measurements. However, the excellent agreement after constant correction (CCC=0.904) demonstrates that simple calibration may render consumer devices suitable for some research contexts.

Population-specific calibration should be performed before using consumer devices in research. Our 62-minute correction factor applies specifically to older Japanese outpatients with cardiac issues and should not be assumed to apply to other populations without validation. Researchers planning to use Fitbit or similar devices should consider conducting preliminary validation studies in their target population to determine appropriate correction factors.

The moderate categorical agreement (κ=0.588) when classifying participants into sedentary time tertiles suggests acceptable performance for epidemiological studies examining associations between categorical sedentary behavior levels and health outcomes. Importantly, all misclassifications occurred between adjacent categories with no extreme misclassifications, indicating that the device maintains relative ordering of individuals. This property may be sufficient for observational studies where precise quantification is less critical than rank-ordering or categorical stratification.

For intervention trials, where detecting within-person change is paramount, our cross-sectional data provide only indirect evidence of suitability. The absence of proportional bias suggests that changes in sedentary time should be measured with similar accuracy regardless of baseline levels, but longitudinal validation is needed to confirm this hypothesis and quantify measurement error for within-person change.

Consumer wearables may offer particular value in large-scale studies where research-grade devices are cost-prohibitive. With appropriate calibration and acknowledgment of limitations, devices like the Fitbit could enable sedentary behavior research in larger, more diverse samples, potentially improving external validity and generalizability of findings.

### Study Limitations

Several limitations warrant consideration and point to important directions for future research.

#### Cross-Sectional Design and Temporal Stability

This was a cross-sectional validation study that assessed agreement between devices at a single time point. While we found strong between-person correlation, the study design does not directly evaluate the device’s ability to track within-person changes over time. Test-retest reliability, day-to-day measurement error, and the minimal detectable change for individual monitoring remain unknown. Future longitudinal studies with repeated measurements over weeks to months are essential to establish whether the device can reliably detect behavioral changes within individuals—a critical requirement for clinical monitoring and intervention trials.

Responsiveness to intervention should be evaluated in prospective studies where sedentary time is intentionally modified through behavioral counseling or structured programs. Such studies would determine whether Fitbit-detected changes correspond to changes measured by research-grade devices and whether these changes predict clinical outcomes such as cardiovascular events, functional capacity, or quality of life.

#### Measurement Variability and Precision

The wide 95% limits of agreement (−11.3 to 135.2 minutes) indicate substantial interindividual variability in measurement error, corresponding to approximately ±73 minutes around the mean bias. The sources of this variability remain unclear but warrant investigation. Potential moderators include:

- Individual movement characteristics: arm movement patterns during activities, gait characteristics, and activity repertoire.
- Physiological factors: body composition (arm circumference, adiposity), cardiovascular functional capacity, and presence of tremor or dyskinesia.
- Device-related factors: wearing position on wrist, tightness of band, and skin temperature affecting sensor performance.
- Behavioral factors: types of activities performed, proportion of time in different postures, and occupation-related demands.

Future research should systematically evaluate these potential moderators to identify subgroups in whom device performance may be particularly strong or weak. Understanding sources of variability could inform personalized correction approaches or help clinicians identify patients for whom alternative monitoring methods may be preferable.

Our Fitbit classification required a step count of exactly zero for a minute to be counted as sedentary. Wrist-worn trackers are known to register “phantom” steps from nonambulatory arm movements such as gesturing, fidgeting, or hand activity performed while seated. Minutes containing such phantom steps would have been classified as nonsedentary even when the participant was in fact sitting, causing the device to under-count sedentary time in those minutes. The net 62-minute overestimation we observed may therefore mask bidirectional misclassification—overestimation when low-movement standing or light activity is misread as sedentary, partially offset by underestimation when seated, phantom-step minutes are misread as active—rather than a purely unidirectional error. The minute-level analyses needed to quantify these opposing components were beyond the scope of the present study and are a priority for future work.

A statistical caveat applies to our cross-validation of the bias correction. Subtracting a constant equal to the mean paired difference will, almost by construction, improve the concordance correlation coefficient, because it removes the location-shift (accuracy) component of disagreement while leaving the precision component unchanged. Furthermore, because the correction has a single parameter, a constant offset estimated as the sample mean, LOOCV re-estimates essentially the same mean at each fold, so the negligible shrinkage we observed reflects the stability of the sample mean within this dataset rather than evidence that the 62-minute correction factor is transportable to other samples or settings. Our cross-validation should therefore be interpreted as an internal-consistency check, not as external validation. Establishing the external validity of the correction factor requires deriving and testing it in independent populations.

#### Sedentary Patterns Beyond Total Time

Our analysis focused on total daily sedentary time within a standardized 12-hour window. We did not examine the validity of detecting sedentary bout duration or breaks in sedentary time—metrics that may be equally or more important for cardiovascular health than total volume. Emerging evidence suggests that prolonged uninterrupted sedentary bouts (eg, >30 or >60 minutes) and the frequency of breaks in sedentary time confer cardiovascular risks independent of total sedentary time. A recent meta-analysis found that replacing one hour of sitting with light-intensity physical activity lowered cardiovascular disease risk by 20%, highlighting the importance of breaking up prolonged sitting.

Future validation work should assess whether consumer devices can accurately identify:

- Bout duration: Can the device distinguish between 6 hours accumulated in 10-minute bouts versus 6 hours in uninterrupted sitting?
- Bout patterns: Can it detect breaks in sedentary time and classify their intensity (standing breaks vs light activity breaks)?
- Temporal patterns: Does the device accurately capture sedentary time distribution across the day (morning vs afternoon vs evening)?

These pattern-based metrics may be more clinically relevant than total volume for cardiovascular risk stratification and intervention targeting [32-36].

#### Generalizability Concerns

The study was conducted in a specific population of Japanese outpatients with cardiac issues with a mean age of approximately 78 (SD 9.0) years, and predominantly with heart failure or ischemic heart disease. Generalizability requires verification across:

- Age groups: younger patients with cardiac issues, middle-aged adults, and very older (>85 years).
- Cardiac conditions: valvular disease, arrhythmias, congenital heart disease, and posttransplant.
- Functional status: NYHA class I vs IV, different exercise capacities.
- Geographic and ethnic populations: Western populations, other Asian countries, and diverse ethnic groups.
- Noncardiac populations: healthy older adults, other chronic conditions (chronic obstructive pulmonary disease, diabetes, and chronic kidney disease).

We could not evaluate whether the measurement bias differed by NYHA functional class because NYHA class was not collected and was not uniformly applicable to all participants. Although musculoskeletal disorders were included as a broad comorbidity category, none of the participants had an upper-limb musculoskeletal disorder documented in the medical records that was expected to affect wrist-worn device measurements. Therefore, the influence of upper-limb impairment on wrist-worn device accuracy could not be evaluated in this cohort.

Device performance may differ substantially across these groups due to varying movement patterns, activity levels, and physiological constraints. The 62-minute correction factor derived from our study should not be applied to other populations without validation.

Although no participant had a documented upper-limb musculoskeletal disorder, 35% (18/51) of the cohort had a musculoskeletal disorder of some kind, and we did not record detailed gait characteristics. Because independent ambulation without an assistive device was an eligibility requirement, walking-aid users were not enrolled, and cane or walker use is therefore unlikely to account for misclassification in the present cohort. Within the enrolled cohort, lower-limb musculoskeletal disease and age-related gait alteration can nonetheless reduce natural arm swing during walking, even in individuals who ambulate independently. Because the wrist-worn device infers activity from arm motion, reduced arm swing during ambulation could cause genuine walking to be registered as sedentary, contributing to the overestimation observed. We were unable to evaluate or adjust for this effect, and future studies should record gait parameters such as arm-swing amplitude and cadence to examine it directly.

The exploratory multivariable analysis of moderators of the bias was underpowered (N=51; model *P*=.12; *R*²=0.15), and the absence of statistically significant moderators carries a high risk of type II error. Nonsignificance does not establish the absence of an effect: the adjusted estimate for heart failure versus ischemic heart disease (+17.4 minutes) had a wide 95% CI (−3.7 to 38.5 minutes) whose upper bound exceeds half of the overall 62-minute bias and is therefore potentially clinically meaningful. Consequently, our data do not establish that a single population-level correction is adequate across clinically important subgroups; subgroup-specific calibration, particularly by primary diagnosis, cannot be excluded and should be evaluated in adequately powered studies.

#### Cultural and Regional Factors

The direction and magnitude of the observed bias may be shaped by culturally specific patterns of daily activity that are common in Japan but less so in Western settings. Traditional floor-based sitting postures (eg, seiza and cross-legged sitting) and many low-intensity household and self-care activities are typically performed with the trunk relatively upright but with limited arm displacement. Because a wrist-worn device infers activity from arm motion, such postures may be misclassified as sedentary even when the participant is not sitting in the conventional sense, plausibly contributing to the overestimation we observed; conversely, validation studies in populations with more arm-dynamic routines have sometimes reported underestimation. Our cohort also differed from many North American and European outpatients with cardiac issues samples in its high mean age (78.4, SD 9 years) and substantial comorbidity burden, both of which tend to reduce ambulatory arm movement. These considerations indicate that neither the direction of bias nor the 62-minute correction factor should be assumed to transfer across cultural and regional contexts. For researchers and clinicians applying consumer wrist-worn devices in other settings, we recommend deriving a population-specific correction factor in a local validation sample, ideally against a posture-based reference such as the activPAL, before using device-derived sedentary time for quantitative purposes, and interpreting cross-population comparisons of absolute sedentary time with caution.

#### Comparator Considerations

We used the ActiveStyle Pro as the comparator, which, while validated against criterion measures (Douglas bag method) for energy expenditure, has not been compared directly with posture-based gold standards such as the activPAL for sedentary behavior classification. The activPAL is a thigh-worn inclinometer or accelerometer widely regarded as a reference method for classifying sitting or lying versus standing and for capturing postural transitions in adult field-based research [37-39].

Some residual measurement error in our comparator device cannot be excluded, which would tend to underestimate the true agreement between the Fitbit and criterion measures. Three-way validation studies comparing consumer devices, movement-based research accelerometers (ActiGraph and ActiveStyle Pro), and posture-based reference standards (activPAL) would strengthen the evidence base and clarify the relative performance of different monitoring approaches.

Our Fitbit sedentary classification relied on the metabolic-equivalent value returned with the Fitbit calories resource, which is itself an output of Fitbit’s proprietary energy-expenditure algorithm. We necessarily assumed that this value corresponds to the same underlying construct as the metabolic-equivalent estimates produced by the ActiveStyle Pro, which were validated against the Douglas bag method. This assumption may not hold, because the two devices estimate metabolic equivalents using different and only partly disclosed algorithms. Part of the observed disagreement may therefore reflect differences between these algorithms, an unmeasured layer of algorithmic confounding, rather than the wrist-versus-waist placement difference alone, and we were unable to isolate this contribution with the available data.

#### Technology Evolution

Device software and algorithms are continuously updated by manufacturers, and our findings pertain specifically to the Fitbit Inspire 3 with the firmware version available during the study period (October 2022 to March 2023). Validation findings may not generalize across different Fitbit models (eg, Charge, Sense, and Versa series) or software versions. Manufacturers frequently update algorithms without notification, potentially altering device performance.

This highlights the need for ongoing surveillance and periodic revalidation as technology evolves. Establishing standardized validation protocols that can be rapidly deployed when new devices or firmware updates are released would help maintain evidence currency.

This study did not include an a priori sample size calculation; consecutive eligible patients enrolled during the study period were analyzed. The sample was therefore not designed to estimate intermethod agreement, and in particular the limits of agreement, with high precision, and the limits of agreement should be interpreted with corresponding caution.

Despite these limitations, this study provides clinically relevant evidence on the measurement characteristics of a widely used consumer-grade wearable in an understudied, high-risk cardiac population and offers a pragmatic foundation for future longitudinal validation and intervention studies in digital cardiovascular care.

### Future Research Directions

Several avenues for future research emerge from this work.

Longitudinal validation studies should evaluate test-retest reliability, minimal detectable change, and responsiveness to behavioral interventions in cardiac populations. Studies should include repeated measurements over weeks to months to characterize within-person measurement error and determine whether devices can reliably detect clinically meaningful changes.

Prospective intervention trials should assess whether Fitbit-detected changes in sedentary time correspond to changes measured by research-grade devices and whether these changes predict clinical outcomes (cardiovascular events, hospital readmissions, functional capacity, and quality of life). Such studies would establish the clinical utility of consumer devices for outcome assessment.

Machine learning and algorithm development studies could explore whether using raw Fitbit accelerometer data (rather than proprietary processed outputs) with population-specific classification algorithms can improve sedentary time accuracy in clinical populations. Recent advances in machine learning may enable personalized calibration approaches that account for individual movement signatures.

Multidevice validation studies would clarify relative performance and inform device selection for different research contexts. Such studies should incorporate a posture-based reference standard such as the activPAL, movement-based research accelerometers such as the ActiGraph and the ActiveStyle Pro, and multiple consumer devices such as the Fitbit, Apple Watch, and Garmin (Garmin Ltd).

Moderator analyses should systematically investigate sources of individual variability in measurement error, including patient characteristics (age, sex, body composition, cardiovascular functional capacity, and comorbidities), device factors (wear location and band tightness), and activity patterns (occupational demands and activity repertoire). Identifying moderators could enable stratified correction approaches or inform patient-device matching.

Pattern-based validation should assess device accuracy for sedentary bout duration, breaks in sedentary time, and temporal distribution patterns—metrics potentially more important than total volume for cardiovascular health.

Implementation research should examine patient and clinician perspectives on wearable-based sedentary behavior monitoring in real-world cardiac care settings, addressing questions of feasibility, acceptability, data interpretation, integration into clinical workflows, and impact on behavior change counseling effectiveness.

Cross-cultural validation should extend this work to diverse geographic and ethnic populations, recognizing that cultural practices (floor-sitting and traditional activities), built environments, and social norms around activity may influence device performance.

### Implications for Clinical Practice and Research

These findings have important implications for both clinical practice and research in digital cardiovascular care. From a clinical perspective, the strong correlation and absence of proportional bias indicate that consumer-grade wrist-worn activity trackers can be used to monitor relative changes in sedentary behavior over time in outpatients with cardiac issues. This property is particularly relevant for remote monitoring, lifestyle counseling, and secondary prevention programs, where trends and behavioral patterns are often more informative than absolute values. However, clinicians should interpret absolute sedentary time estimates with caution, as wrist-worn devices may systematically overestimate sedentary behavior in older patients with cardiac issues. Applying a simple population-specific correction, as demonstrated in this study, may help contextualize tracker-derived metrics when setting behavioral targets or evaluating intervention effects.

From a research perspective, our results suggest that consumer-grade wrist-worn devices should not be used interchangeably with research-grade accelerometers for precise quantification of sedentary time without appropriate calibration. The moderate uncorrected concordance observed in this study indicates that such devices may be suboptimal as primary outcome measures when absolute sedentary time is required. Nevertheless, the substantial improvement in agreement after constant correction and the acceptable categorical agreement support their potential utility for large-scale observational studies, ranking-based analyses, and intervention trials focused on within-person change. Importantly, these findings underscore the necessity of population-specific validation and calibration when deploying consumer wearables in clinical research, particularly in older or functionally limited populations.

### Conclusions

In older Japanese outpatients with cardiac issues, the Fitbit Inspire 3 systematically overestimated sedentary time by approximately 62 minutes within a standardized 12-hour daytime window compared with the waist-worn ActiveStyle Pro comparator, with significant fixed bias but no proportional bias. The between-device correlation was strong (*r*=0.905), and a simple constant correction improved concordance from moderate (CCC=0.71) to high; cross-validation indicated that this in-sample improvement was internally stable (out-of-sample CCC=0.90). In an exploratory analysis, the bias was not significantly moderated by sex, age, primary diagnosis, or musculoskeletal comorbidity, although this analysis was underpowered and a clinically relevant difference, particularly by primary diagnosis, cannot be excluded. Of greater practical importance, the wide limits of agreement indicate substantial interindividual variability that constrains the device’s precision for individual-level measurement, and categorical agreement was only moderate (κ=0.59). These findings indicate that, in this population, a bias-corrected consumer wrist-worn tracker can provide group-level estimates and relative comparisons of sedentary time, but should not be treated as interchangeable with a research-grade comparator for precise individual quantification. Because the correction factor is specific to this population, device generation, and 12-hour measurement window, it should not be transferred to other settings without independent validation.

## Abbreviations

ACE: angiotensin-converting enzyme
ARB: angiotensin receptor blocker
ARNI: angiotensin receptor–neprilysin inhibitor
CAD: coronary artery disease
CCC: concordance correlation coefficient
CVD: cardiovascular disease
HCN: hyperpolarization-activated cyclic nucleotide
LOOCV: leave-one-out cross-validation
LPA: light-intensity physical activity
MET: metabolic equivalent
MVPA: moderate to vigorous physical activity
NYHA: New York Heart Association
RR: relative risk
SB: sedentary behavior
SGLT2: sodium-glucose cotransporter-2

## References

[1] Tremblay MS, Aubert S, Barnes JD (2017). Sedentary Behavior Research Network (SBRN) - Terminology Consensus Project process and outcome. Int J Behav Nutr Phys Act.

[2] Young DR, Hivert MF, Alhassan S (2016). Sedentary behavior and cardiovascular morbidity and mortality: a science advisory from the American Heart Association. Circulation.

[3] Onagbiye S, Guddemi A, Baruwa OJ (2024). Association of sedentary time with risk of cardiovascular diseases and cardiovascular mortality: a systematic review and meta-analysis of prospective cohort studies. Prev Med.

[4] Ajufo E, Kany S, Rämö JT (2025). Accelerometer-measured sedentary behavior and risk of future cardiovascular disease. J Am Coll Cardiol.

[5] LaMonte MJ, LaCroix AZ, Nguyen S (2024). Accelerometer-measured physical activity, sedentary time, and heart failure risk in women aged 63 to 99 years. JAMA Cardiol.

[6] Bull FC, Al-Ansari SS, Biddle S (2020). World Health Organization 2020 guidelines on physical activity and sedentary behaviour. Br J Sports Med.

[7] Naito K, Izawa KP, Maeda N, Kasai Y, Iwama H (2024). Characteristics of physical activity and sedentary behavior in patients undergoing outpatient cardiac rehabilitation. Sci Rep.

[8] Izawa KP, Oka K (2024). Focusing on sedentary behavior in comprehensive cardiac rehabilitation. Circ Rep.

[9] Izawa KP, Kitamura M, Ogura A (2025). Impact of sedentary time and physical function on health-related quality of life in hospitalized patients with cardiovascular disease. CJC Open.

[10] van Bakel BMA, Kroesen SH, Bakker EA (2023). Effectiveness of an intervention to reduce sedentary behaviour as a personalised secondary prevention strategy for patients with coronary artery disease: main outcomes of the SIT LESS randomised clinical trial. Int J Behav Nutr Phys Act.

[11] Patterson K, Davey R, Keegan R (2023). Testing the effect of a smartphone app on hospital admissions and sedentary behavior in cardiac rehabilitation participants: ToDo-CR randomized controlled trial. JMIR Mhealth Uhealth.

[12] Saleh ZT, Aslanoğlu A, Almagharbeh WT (2025). Reducing sedentary behavior improves depressive symptoms among patients with heart failure enrolled in a home-based mobile health app cardiac rehabilitation. J Nurs Scholarsh.

[13] Oshima Y, Kawaguchi K, Tanaka S (2010). Classifying household and locomotive activities using a triaxial accelerometer. Gait Posture.

[14] Evenson KR, Goto MM, Furberg RD (2015). Systematic review of the validity and reliability of consumer-wearable activity trackers. Int J Behav Nutr Phys Act.

[15] Germini F, Noronha N, Borg Debono V (2022). Accuracy and acceptability of wrist-wearable activity-tracking devices: systematic review of the literature. J Med Internet Res.

[16] Giurgiu M, Ketelhut S, Kubica C (2023). Assessment of 24-hour physical behaviour in adults via wearables: a systematic review of validation studies under laboratory conditions. Int J Behav Nutr Phys Act.

[17] Hajj-Boutros G, Landry-Duval MA, Comtois AS, Gouspillou G, Karelis AD (2023). Wrist-worn devices for the measurement of heart rate and energy expenditure: a validation study for the Apple Watch 6, Polar Vantage V and Fitbit Sense. Eur J Sport Sci.

[18] Delobelle J, Lebuf E, Dyck DV (2024). Fitbit’s accuracy to measure short bouts of stepping and sedentary behaviour: validation, sensitivity and specificity study. Digit Health.

[19] Rowlands AV, Olds TS, Hillsdon M (2014). Assessing sedentary behavior with the GENEActiv: introducing the sedentary sphere. Med Sci Sports Exerc.

[20] Bailey DP, Ahmed I, Cooper DL (2025). Validity of a wrist-worn consumer-grade wearable for estimating energy expenditure, sedentary behaviour, and physical activity in manual wheelchair users with spinal cord injury. Disabil Rehabil Assist Technol.

[21] Bland JM, Altman DG (1986). Statistical methods for assessing agreement between two methods of clinical measurement. Lancet.

[22] Lin LIK (1989). A concordance correlation coefficient to evaluate reproducibility. Biometrics.

[23] Cohen J (1960). A coefficient of agreement for nominal scales. Educ Psychol Meas.

[24] Collins JE, Yang HY, Trentadue TP, Gong Y, Losina E (2019). Validation of the Fitbit Charge 2 compared to the ActiGraph GT3X+ in older adults with knee osteoarthritis in free-living conditions. PLoS One.

[25] Gomersall SR, Ng N, Burton NW, Pavey TG, Gilson ND, Brown WJ (2016). Estimating physical activity and sedentary behavior in a free-living context: a pragmatic comparison of consumer-based activity trackers and ActiGraph accelerometry. J Med Internet Res.

[26] Carpenter C, Yang CH, West D (2021). A comparison of sedentary behavior as measured by the Fitbit and ActivPAL in college students. Int J Environ Res Public Health.

[27] Redenius N, Kim Y, Byun W (2019). Concurrent validity of the Fitbit for assessing sedentary behavior and moderate-to-vigorous physical activity. BMC Med Res Methodol.

[28] Salim A, Brakenridge CJ, Lekamlage DH (2024). Detection of sedentary time and bouts using consumer-grade wrist-worn devices: a hidden semi-Markov model. BMC Med Res Methodol.

[29] Yano S, Koohsari MJ, Shibata A, Ishii K, Mavoa S, Oka K (2019). Assessing physical activity and sedentary behavior under free-living conditions: comparison of active style pro HJA-350IT and ActiGraphTM GT3X. Int J Environ Res Public Health.

[30] Kurita S, Yano S, Ishii K (2017). Comparability of activity monitors used in Asian and Western-country studies for assessing free-living sedentary behaviour. PLoS One.

[31] Sasai H (2017). Assessing sedentary behavior using wearable devices: an overview and future directions. J Phys Fit Sports Med.

[32] Wu J, Fu Y, Chen D (2023). Sedentary behavior patterns and the risk of non-communicable diseases and all-cause mortality: a systematic review and meta-analysis. Int J Nurs Stud.

[33] Swartz AM, Steinbrink GM, Strath SJ, Mitra T, Morelli WA (2025). A systematic review of the effects of breaking up/interrupting sedentary behavior on health outcomes among community-dwelling adults 60+ years. J Aging Phys Act.

[34] Liang YY, He Y, Huang P (2025). Accelerometer-measured physical activity, sedentary behavior, and incidence of macrovascular and microvascular events in individuals with type 2 diabetes mellitus and prediabetes. J Sport Health Sci.

[35] Farrahi V, Collings PJ, Oussalah M (2024). Deep learning of movement behavior profiles and their association with markers of cardiometabolic health. BMC Med Inform Decis Mak.

[36] Hyde ET, Bandoli GE, Zou J (2025). Prospective associations between accelerometer-measured physical activity, sedentary behavior, and healthy longevity: the Women’s Health Accelerometry Collaboration. J Gerontol A Biol Sci Med Sci.

[37] Grant PM, Ryan CG, Tigbe WW, Granat MH (2006). The validation of a novel activity monitor in the measurement of posture and motion during everyday activities. Br J Sports Med.

[38] O’Brien MW, Wu Y, Petterson JL, Bray NW, Kimmerly DS (2022). Validity of the ActivPAL monitor to distinguish postures: a systematic review. Gait Posture.

[39] Edwardson CL, Winkler EAH, Bodicoat DH (2017). Considerations when using the activPAL monitor in field-based research with adult populations. J Sport Health Sci.

